# Direct prediction of antimicrobial resistance in *Pseudomonas aeruginosa* by metagenomic next-generation sequencing

**DOI:** 10.3389/fmicb.2024.1413434

**Published:** 2024-06-06

**Authors:** Lichao Cao, Huilin Yang, Zhigang Huang, Chang Lu, Fang Chen, Jiahao Zhang, Peng Ye, Jinjin Yan, Hezi Zhang

**Affiliations:** ^1^Shenzhen Nucleus Gene Technology Co., Ltd., Shenzhen, Guangdong Province, China; ^2^Department of Laboratory Medicine, Peking University Shenzhen Hospital, Shenzhen, Guangdong Province, China

**Keywords:** *Pseudomonas aeruginosa*, multi-drug resistance, anti-microbial resistance, metagenomics next-generation sequencing, resistance prediction model

## Abstract

**Objective:**

*Pseudomonas aeruginosa* has strong drug resistance and can tolerate a variety of antibiotics, which is a major problem in the management of antibiotic-resistant infections. Direct prediction of multi-drug resistance (MDR) resistance phenotypes of *P. aeruginosa* isolates and clinical samples by genotype is helpful for timely antibiotic treatment.

**Methods:**

In the study, whole genome sequencing (WGS) data of 494 *P. aeruginosa* isolates were used to screen key anti-microbial resistance (AMR)-associated genes related to imipenem (IPM), meropenem (MEM), piperacillin/tazobactam (TZP), and levofloxacin (LVFX) resistance in *P. aeruginosa* by comparing genes with copy number differences between resistance and sensitive strains. Subsequently, for the direct prediction of the resistance of *P. aeruginosa* to four antibiotics by the AMR-associated features screened, we collected 74 *P. aeruginosa* positive sputum samples to sequence by metagenomics next-generation sequencing (mNGS), of which 1 sample with low quality was eliminated. Then, we constructed the resistance prediction model.

**Results:**

We identified 93, 88, 80, 140 AMR-associated features for IPM, MEM, TZP, and LVFX resistance in *P. aeruginosa*. The relative abundance of AMR-associated genes was obtained by matching mNGS and WGS data. The top 20 features with importance degree for IPM, MEM, TZP, and LVFX resistance were used to model, respectively. Then, we used the random forest algorithm to construct resistance prediction models of *P. aeruginosa*, in which the areas under the curves of the IPM, MEM, TZP, and LVFX resistance prediction models were all greater than 0.8, suggesting these resistance prediction models had good performance.

**Conclusion:**

In summary, mNGS can predict the resistance of *P. aeruginosa* by directly detecting AMR-associated genes, which provides a reference for rapid clinical detection of drug resistance of pathogenic bacteria.

## 1 Introduction

*Pseudomonas aeruginosa* is a Gram-negative opportunistic bacterium that causes a variety of acute and chronic infections in humans. Due to its increasing incidence, high treatment difficulty, and high fatality rate, it is a major problem and focus of clinical treatment (Fernández-Barat et al., 2017; Reynolds and Kollef, 2021). The global burden report of bacterial anti-microbial resistance (AMR) in 2019 pointed out that AMR poses a major threat to human health worldwide, especially the resistance of *P. aeruginosa* to multiple antibiotics (Murray et al., 2022). With a relatively large bacterial genome, *P. aeruginosa* has good tolerance and adaptability to various environments, and has natural resistance to a variety of antibiotics (Klockgether et al., 2011), among which difficult-to-treat resistance *P. aeruginosa* (DTR-PA) urgently needs research and development of new antibiotics to deal with opportunistic infections caused by DTR-PA (Qin et al., 2022).

Rapid detection and elucidation of resistance mechanisms are essential for timely antibiotic treatment and monitoring of multi-drug resistance (MDR) *P. aeruginosa*. Nowadays, whole genome sequencing (WGS) has become an advanced method for detecting AMR (Maladan et al., 2021), while the clinical application of WGS is limited by high cost, high sample requirements, and technical analytical hurdles. The application of antibiotic susceptibility testing (AST) in the diagnosis of antimicrobial-resistant pathogens and their antibiogram is time-consuming, cumbersome operation and has a low positive rate, which cannot meet the clinical needs (Shanmugakani et al., 2020; Hu and Zhao, 2023). Recently, metagenomics next-generation sequencing (mNGS) technology enables the identification of pathogens and AMR-associated genes directly from clinical samples based on its ability of the rapid diagnosis of unexplained infections (Yan et al., 2021; Liu et al., 2023). Therefore, considering the complex resistance mechanisms and the high prevalence of variant-driven resistance of DTR-PA, it is feasible to predict resistance phenotypes of DTR-PA by detecting AMR-associated genes via mNGS.

In this study, mNGS was used to directly predict resistance to multiple antibiotics in *P. aeruginosa*, such as imipenem (IPM), meropenem (MEM), piperacillin/tazobactam (TZP), and levofloxacin (LVFX). First, AMR-associated genes of *P. aeruginosa* were screened according to the resistance genotype and phenotype of WGS and AST data. Then, we matched the AMR-associated genes from mNGS data of *P. aeruginosa* in clinical samples with the above genes to obtain the relative abundance of genes. Finally, the direct resistance prediction models for *P. aeruginosa* were constructed using random forest (RF) based on the relative abundance of AMR-associated genes.

## 2 Materials and methods

### 2.1 Genome and AMR phenotype collection of *Pseudomonas aeruginosa* strains

The NCBI NDARO database^1^ and reference (Liu et al., 2023) were performed for searching *P. aeruginosa* strains with both whole genome sequences and unambiguous resistance phenotypes to IPM, MEM, TZP, and LVFX. Referencing the previous research (Hu and Zhao, 2023), low quality genomes were eliminated using a customized criterion developed with reference to NCBI genome exclusion rules. See the Supplementary Figure S1 for the filtering rules. After this filtering step, a total of 494 *P. aeruginosa* whole genomes were obtained, including 394 *P. aeruginosa* strains from NCBI NDARO database and 100 *P. aeruginosa* strains from the referenced study (Liu et al., 2023). Detailed information on the genomes of *P. aeruginosa* strains is displayed in Supplementary Table S1.

### 2.2 Curation of the *Pseudomonas aeruginosa* reference database and mapping

Prodigal v2.6.3 was used to predict the genes of the collected genome, and compare the predicted genes with eggNOG-mapper v2 (Cantalapiedra et al., 2021) to get the gene name; finally the gene copy number was obtained from knowing number of gene names in bacterial genome (Supplementary Table S2). Subsequently, R language function t.test was applied to perform differential analysis on the resistance and sensitive groups of the same antibiotic. The obtained *p* values were corrected to *q* values using the R language function p.adjust (holm method). Genes with *q*-values <0.05 were regard as AMR-associated genes.

### 2.3 Patients, samples, and processing

A total of 74 *P. aeruginosa* positive sputum samples from 59 clinical patients were collected retrospectively from Peking University Shenzhen Hospital from February 2023 to September 2023. Our study has obtained the oral informed consent from all subjects, and approved by the Medical Ethics Committee of Peking University Shenzhen Hospital.

### 2.4 DNA extraction, sequencing, and quality control

DNA was extracted from each sample by using the FastPure Host Removal and Microbiome DNA Isolation Kit (Catalog No. DC501, Vazyme, China). Metagenomic sequencing was done on the MGI-200 platform (BGI, Shenzhen, China) (50 bp of single-end reads for all samples). The real resistant samples and sensitive samples were combined respectively, and then Seqtk (v1.4) was used to randomly extract sequences from the real samples in equal proportions to obtain the simulated samples (Shen et al., 2016). Subsequently, the original sequencing file was filtered with fastp (v0.19.4) (Chen et al., 2018) to filter out low-quality sequences, and then the host decontamination was used by Bowtie2 (v2.3.5) with referencing human genome GRCh38 (Langmead and Salzberg, 2012).

### 2.5 Construction of predict model

As mentioned above, we obtained 93, 88, 80, and 140 AMR-associated genes for IPM, MEM, TZP, and LVFX, respectively. Then, we used RF to select the top 20 genes with the highest contribution to the model as features. Subsequently, top 20 AMR-associated gene were trained by three machine learning methods, including RF, logistic regression, and support vector machine (SVM), to build resistance prediction models. All the models were carried out with the “caret 6.0.86” package (random forest version 4.6.14). Real resistance samples: real sensitive samples: simulated resistance samples: simulated sensitive samples were selected in equal proportion. The samples were randomly divided into the training set and the test set in a 1:1 ratio.

### 2.6 Analysis of the relative abundance of AMR-associated genes

Wilcoxon rank-sum test (*p* < 0.05) was used to compare the relative abundance of AMR-associated genes between resistance samples and sensitive samples. The R package “pheatmap 1.0.12” was used to draw a heatmap of the relative abundance of AMR-associated genes between resistance samples and sensitive samples. Violin diagram showed the top three AMR-associated genes between resistance samples and sensitive samples by the R Package “ggpubr 0.6.0.”

## 3 Results

### 3.1 Screening of key AMR-associated genes of *Pseudomonas aeruginosa*

**Figure 1:**
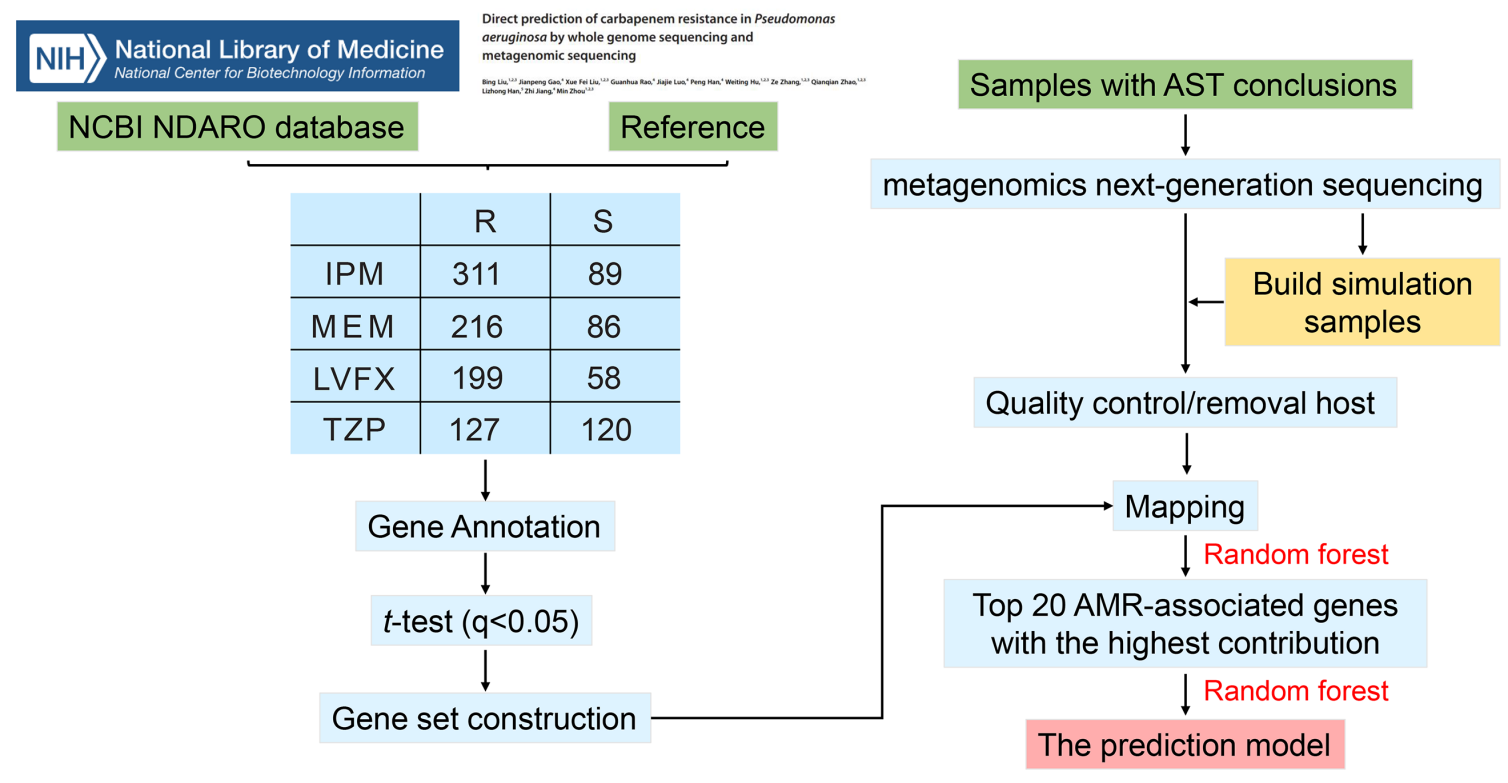
The flowchart of this study.

The workflow of our study was presented in Figure 1. There was 400, 302, 247, and 257 *P. aeruginosa* strains resistant or sensitive to IPM, MEM, TZP, and LVFX, respectively. The total resistance rates of these strains to IPM, MEM, TZP, and LVFX were 77.75% (311/400), 71.52% (216/302), 51.42% (127/247), and 77.43% (199/257), respectively. Regarding genetic characteristics, we obtained genes with significant differences in copy number between resistant and sensitive strain groups using *t*-test analysis. These AMR-associated genes with IPM, MEM, TZP, and LVFX resistance identified in *P. aeruginosa* isolates were summarized in Supplementary Table S3. In total, 93 gene associated with IPM resistance were obtained in *P. aeruginosa* isolates, including yebS, ymdF, stbD, hcnA, ydhM, yfjR, clsB, puuP3, yedA, moeB, katE, mmlH, MA20_16685, sod22, MA20_16,375, treS, MA20_16485, hcnB, spxB, and prhI, etc. Moreover, there were 88 AMR-associated genes with MEM resistance, 80 AMR-associated genes with TZP resistance and 140 AMR-associated genes with LVFX resistance in *P. aeruginosa*. See Supplementary Table S3 for detailed information. Then, we took the intersection of all AMR-associated genes, where HA62_05660, merA, merP were common AMR-associated genes with four antibiotics resistance (Supplementary Figure S2; Supplementary Table S4). Subsequently, we chose top 20 AMR-associated genes with the highest contribution to the models as features by using random forest in the prediction models for IPM, MEM, TZP, and LVFX, respectively (Supplementary Table S5).

### 3.2 Selection of machine learning method

**Figure 2:**
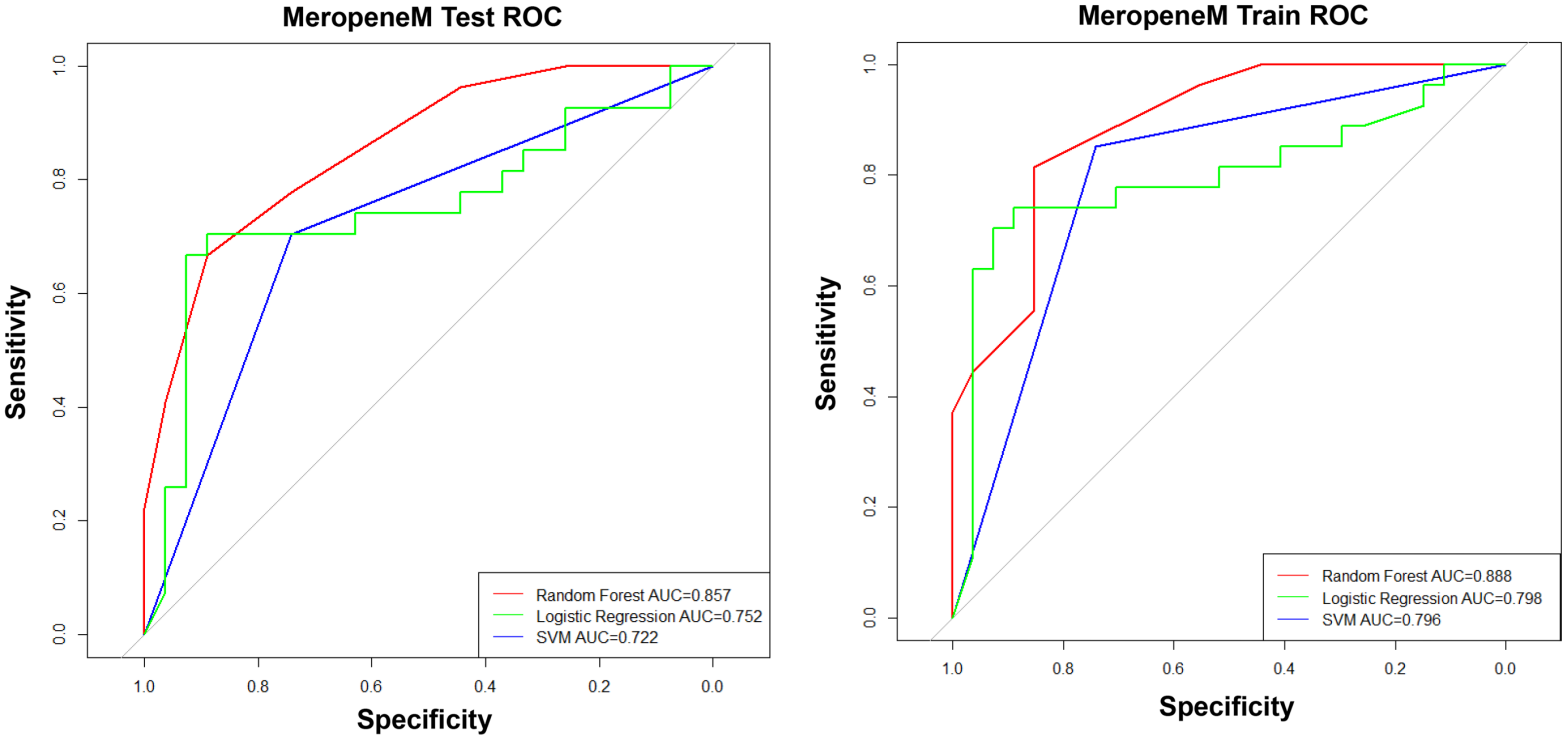
Performance of prediction models constructed by different machine learning methods.

To obtain an appropriate machine learning method, we compared the performance of prediction models for the MEM resistance constructed by RF, logistic regression, and SVM. The results showed that the prediction models built by RF had the best performance of the prediction models with the maximum area under the curve (AUC > 0.85 in both the test cohort and training cohort) (Figure 2). Therefore, RF was performed for the construction of prediction models.

### 3.3 Direct prediction of antibiotic resistance for *Pseudomonas aeruginosa* by mNGS

**Figure 3:**
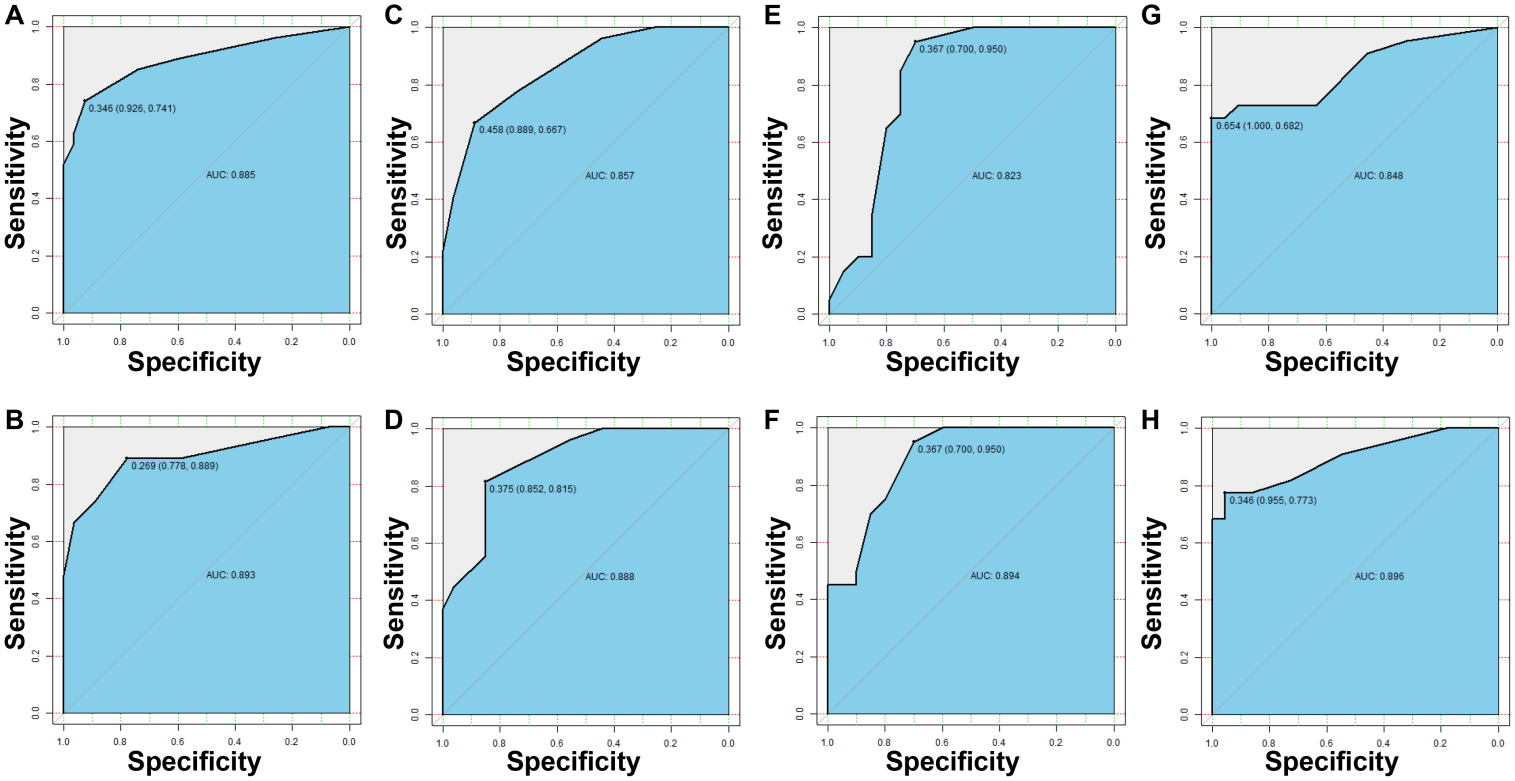
The resistance prediction models of *Pseudomonas aeruginosa*. The IPM resistance prediction of *P. aeruginosa* in the test set **(A)** and the training set **(B)**. The MEM resistance prediction of *P. aeruginosa* in the test set **(C)** and the training set **(D)**. The TZP resistance prediction of *P. aeruginosa* in the test set **(E)** and the training set **(F)**. The LVFX resistance prediction of *P. aeruginosa* in the test set **(G)** and the training set **(H)**.

To directly predict resistance of *P. aeruginosa* to IPM, MEM, TZP, and LVFX from clinical specimens, we evaluated the applicability of mNGS to detect key AMR-associated genes. We collected 74 *P. aeruginosa* positive sputum samples and sent them for mNGS sequencing, of which one sample was eliminated due to quality problems. Subsequently, the AMR-associated genes in mNGS data were mapped with AMR-associated genes selected by machine learning to obtain their relative abundance. Then, according to the RF algorithms, we divided the samples into a test cohort and a training cohort in a 1:1 ratio to create the resistance prediction models for IPM, MEM, TZP, and LVFX, respectively. In the test cohort, the AUC of the IPM resistance prediction model was 0.885, with a sensitivity of 0.741 and a specificity of 0.926 (Figure 3A). The AUC of the MEM resistance prediction model was 0.857, with a sensitivity of 0.667 and a specificity of 0.889 (Figure 3C). The AUC of TZP and LVFX reached 0.823 (a specificity of 0.7 and sensitivity of 0.95), 0.848 (a specificity of 1 and a sensitivity of 0.682), respectively (Figures 3E,G). In the training set, the AUCs of the IPM, MEM, TZP, and LVFX resistance prediction models were 0.893, 0.888, 0.894, and 0.896, respectively (Figures 3B,D,F,H). Taken together, the resistance prediction models for IPM, MEM, TZP, and LVFX based on mNGS sequencing had good diagnostic performance.

### 3.4 Comparison of the relative abundance of the resistance genes between resistance and sensitive samples

**Figure 4:**
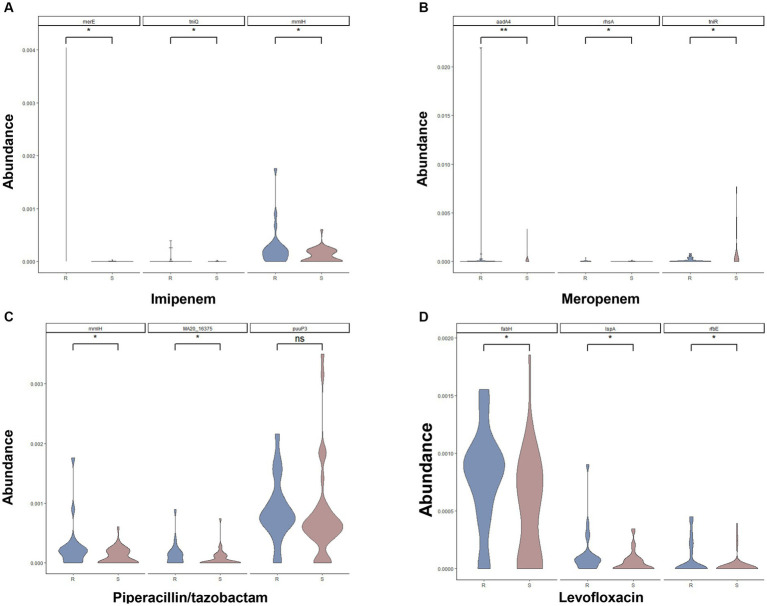
Comparison of the relative abundance of top three AMR-associated genes for the IPM **(A)**, MEM **(B)**, TZP **(C)**, and LVFX **(D)** resistance of *Pseudomonas aeruginosa* between resistant samples and sensitive samples.

We further compared the relative abundance of the resistance genes between resistance samples and sensitive samples. Supplementary Figures S3–S6 indicated the relative abundance of 20 AMR-associated genes between resistance samples and sensitive samples from IPM, MEM, TZP, and LVFX, respectively. The violin diagrams showed the relative abundance of the top three genes with most significant difference in resistance samples and sensitive samples (Figures 4A–D). For example, for the IPM resistance prediction model, the abundance of merE, tniQ, and mmIH in resistant samples was higher than in sensitive samples (*p* < 0.05, Figure 4A), suggesting that these genes were positively associated with IPM resistance. For the MEM resistance prediction model, the abundance of aadA4, rhsA, and tniR in resistant samples was higher than in sensitive samples (*p* < 0.05, Figure 4B), indicating these genes were positively associated with MEM resistance. For the TZP resistance prediction model, compared with the sensitive samples, the abundance of mmH and MA20-16375 was increased in resistant samples (*p* < 0.05, Figure 4C). Similarly, the abundance of fabH, IspA, and rfbE was higher in resistant samples (*p* < 0.05, Figure 4D) in the LVFX resistance prediction model, comparing to sensitive samples. These findings demonstrated the relative abundance of the AMR-associated genes between resistance samples and sensitive samples could influence the resistance or sensitivity of *P. aeruginosa* to antibiotics.

## 4 Discussion

In recent years, due to the abuse of antibiotics, the resistance of *P. aeruginosa* has increased greatly, resulting in MDR and extensive drug resistance strains often appear in clinical treatment, which brings great difficulties to the clinical treatment of patients (Lister et al., 2009; Chegini et al., 2020; Tenover et al., 2022). However, the complex mechanism of drug resistance hindered genotype-to-phenotype prediction of *P. aeruginosa* (Poole, 2002; Fajardo et al., 2008). In view of this, we explored the applicability of WGS and mNGS in the direct prediction of IPM, MEM, TZP, and LVFX resistance of *P. aeruginosa*. Briefly, using the available WGS data of *P. aeruginosa*, 20 AMR-associated genes with IPM, MEM, TZP, and LVFX resistance were identified, respectively. Subsequently, we constructed IPM, MEM, TZP, and LVFX resistance prediction models based on the results of mNGS sequencing. The AUCs of IPM, MEM, TZP, and LVFX resistance prediction models were all greater than 0.8, indicating that our prediction models had good performance in predicting resistance of *P. aeruginosa* to antibiotics.

Several previous studies have explored the performance of WGS in predicting *P. aeruginosa* resistance phenotypes (Khaledi and Weimann, 2020; Kim and Greenberg, 2020; Cortes-Lara et al., 2021). Most of these studies focused on DNA sequences of gene presence or absence and gene variant (Hyun and Kavvas, 2020; Kim and Greenberg, 2020; Cortes-Lara et al., 2021). However, the model-building processes of the models constructed are cumbersome. Additionally, the predictive value of AMR-related gene signatures in *P. aeruginosa* needs to be further verified. The drug resistance mechanism of *P. aeruginosa* mainly involved the outer membrane channel protein OprD gene (Chevalier et al., 2017), the aminoglycoside modifying enzyme gene (Thacharodi and Lamont, 2022), the β-lactam coding gene (Doss et al., 2004), and the 16S rRNA methylase gene (Yokoyama et al., 2003). Liu et al. (2023) have constructed the resistance prediction models for *P. aeruginosa* by detecting deletion or mutation sites of these genes. In our study, genes with significant copy number differences between resistant strains and sensitive strains were regarded as candidate AMR-associated genes associated with IPM/MEM/TZP/LVFX resistance. The candidate AMR-associated genes were used to build corresponding resistance prediction models by RF algorithms. For example, AMR-associated genes in TZP resistance prediction model are ymdF, stbD, hcnA, yebS, etc. The ymdF played a role in flagellum-dependent motility regulation of *P. aeruginosa* (Oguri et al., 2019). Flagellum motility plays an active role in many biological functions of bacteria, such as the formation of bacteria-host symbiosis, pathogenicity, and antibiotic resistance (Raina et al., 2019; Wadhwa and Berg, 2022). In the pathogenic process of *Acinetobacter baumannii*, flagellar dysfunction can significantly reduce its virulence (Corral et al., 2021). Moreover, ymdF as PA2146 homologs contributes to biofilm formation and drug tolerance in *Escherichia coli*, *Klebsiella pneumoniae*, and *P. aeruginosa* (Kaleta et al., 2022; Kaleta and Sauer, 2023), indicating ymdF plays an important role in TZP resistance for *P. aeruginosa*. In addition, stbD is a key component of IncFIB-4.1/4.2 single-replicon plasmids that influences bacterial resistance to antibiotics (Xu et al., 2022). The toxin-antitoxin system is widely present in pathogenic microorganisms, which promotes the formation of MDR bacteria by regulating several important cellular processes in cells (Bernard and Couturier, 1992; Lewis, 2010). stbD/E-pEP36 has reported to be a functional toxin-antitoxin hybrid module (Unterholzner et al., 2013), indicating stbD/E-pEP36 contributed to *P. aeruginosa* resistance to TZP. The high pathogenicity of *P. aeruginosa* is attributed to its production of multiple virulence factors and its resistance to several antimicrobials, among which sodium hypochlorite (NaOCl) is widely used because of its strong antibacterial effect. However, hydrogen cyanide derived from hcnA acts as a scavenger molecule that can quench the toxic effects of NaOCl, thereby contributing to *P. aeruginosa* resistance to NaOCl (da Cruz Nizer et al., 2023). The above information indicated that these AMR-associated genes were significant for *P. aeruginosa* resistance to TZP. Besides, uspA is a common AMR-associated gene in IPM and MEM resistance models. Universal stress proteins (USPs) are generally overexpressed in a variety of pathogens under various environmental stresses, among which uspA has been identified as a potential drug target against MDR*-E. coli* (Kvint et al., 2003). Furthermore, inhibition of UspA function in bacteria can improve the MDR resistance problem of bacteria (Bandyopadhyay and Mukherjee, 2022), suggesting the management of uspA could affect carbapenem resistance of *P. aeruginosa*. FabH inhibitors are new targets and novel antimicrobial agents to overcome bacterial resistance (Castillo and Pérez, 2008). In our study, fabH is an AMR-associated genes with high contribution in LVFX resistance model. FabH is a key enzyme responsible for fatty acid biosynthesis, which is essential for many pathogens (Yuan et al., 2012). According to research findings, the activity ratio of fabH/fabF was the main determinant of antibiotic susceptibility of pathogens (Parsons et al., 2015), suggesting that focusing on the fabH/fabF activity ratio in *P. aeruginosa* could predict resistance to LVFX.

Different from other drug resistance models (Khaledi and Weimann, 2020; Hu and Zhao, 2023; Liu et al., 2023), we have screened AMR-associated genes by comparing the copy number of genes in the antibiotic resistance group and the sensitive group. Metagenomic studies have shown that copy number variation in the human microbiome is common and affects human health (Greenblum et al., 2015; Zeevi et al., 2019). The study have found that an increase in the copy number of antibiotic resistance genes (ARGs) on the multi-copy plasmid promoted the high expression of ARGs, thereby increasing drug resistance (Yao et al., 2022). Several recent studies have reported the relationship between gene duplication and bacterial resistance in clinical antibiotic-resistant strains by measuring copy number of resistant gene (Duvernay et al., 2011; McGann et al., 2014; Chirakul et al., 2019; Anderson et al., 2020). Besides, a new research demonstrated that ARG duplication could be an effective mechanism for the evolution of antibiotic resistance (Maddamsetti et al., 2024). These above studies indicated that measuring copy number changes of ARG in antibiotic-resistant isolates is very important for studying microbial resistance. In our study, AMR-associated genes in *P. aeruginosa* were identified by comparing genes with copy number differences between resistance strains and sensitive strains. Subsequently, four resistance models constructed by AMR-associated genes had good performance, which provide a new molecular resistance analysis tool for predicting antimicrobial resistance.

The detection of antimicrobial resistance mainly relies on the traditional drug susceptibility test, which has some shortcomings such as time-consuming and inappropriate empirical treatment. Therefore, a more rapid antibiotic sensitivity test is urgently needed to effectively reduce antibiotic resistance and improve clinical treatment. Given the widespread use of mNGS and its ability to directly identify AMR-associated genes from a variety of clinical samples, mNGS holds great potential for rapid detection of AMR (Hoang et al., 2021; Ruppé et al., 2022). Hu and Zhao (2023) reported that the average reporting time of the NGS-based AST for clinical samples was 19.1 h, which effectively shortened the reporting time of traditional drug sensitivity detection technology. In our study, we identified AMR-associated genes in *P. aeruginosa* by comparing genes with copy number differences between resistance strains and sensitive strains. Then, *P. aeruginosa* positive sputum samples were sequenced by mNGS method, and AMR-associated genes in mNGS were mapped with AMR-associated genes selected by machine learning to obtain their relative abundance, thereby constructing the resistance prediction models with great performance (AUCs >0.8). Here, we have effectively demonstrated the great applicability of mNGS in the prediction of *P. aeruginosa* to four commonly used antibiotics (IPM, MEM, TZP, and LVFX) based on key AMR-associated features selected and prediction models constructed.

There are certain challenges with the mNGS method. First, the high cost of mNGS is a great challenge for the future extensive application of mNGS. Second, the comprehensive application of mNGS is limited by high host DNA content, nucleic acid contamination, complex interpretation of mNGS data, and the depth of sequencing. Third, as a new detection method, the positive result of mNGS represents the nucleic acid fragment of a certain pathogen detected in clinical specimens.

## 5 Conclusion

In this study, we first proposed genes with significant copy number differences between resistant strains and sensitive strains were associated with resistance of *P. aeruginosa*. These key AMR-associated genes and the prediction models can be used for mNGS to directly predict the resistance of *P. aeruginosa* in clinical samples, which have guiding significance for the clinical management of DTR-PA.

## Data availability statement

The datasets presented in this study can be found in online repositories. The names of the repository/repositories and accession number(s) can be found at: https://www.ncbi.nlm.nih.gov/, PRJNA1060663.

## Ethics statement

The studies involving humans were approved by Ethics Committee of Peking University Shenzhen Hospital. The studies were conducted in accordance with the local legislation and institutional requirements. Written informed consent for participation was not required from the participants or the participants’ legal guardians/next of kin because all patients included provided oral informed consent.

## Author contributions

LC: Conceptualization, Data curation, Formal analysis, Writing – original draft. HY: Conceptualization, Data curation, Formal analysis, Writing – original draft. ZH: Conceptualization, Project administration, Writing – review & editing. CL: Data curation, Methodology, Writing – review & editing. FC: Data curation, Methodology, Writing – review & editing. JZ: Data curation, Methodology, Writing – review & editing. PY: Data curation, Formal analysis, Writing – review & editing. JY: Data curation, Formal analysis, Writing – review & editing. HZ: Conceptualization, Project administration, Resources, Writing – review & editing.

## Footnotes

^1^ https://www.ncbi.nlm.nih.gov/pathogens/antimicrobial-resistance/

## Supplementary material

SUPPLEMENTARY TABLE S1 Detailed information of *P. aeruginosa* included in this study.

SUPPLEMENTARY TABLE S2 The information about the gene copy number. The IDs marked in green were from the referenced study, and the IDs marked in purple were from the NCBI NDARO database.

SUPPLEMENTARY TABLE S3 The AMR-associated genes with *q* < 0.05 for the IPM, MEM, TZP, and LVFX resistance of *P. aeruginosa*.

SUPPLEMENTARY TABLE S4 The common AMR-associated genes in four antibiotics.

SUPPLEMENTARY TABLE S5 Top 20 AMR-associated genes with the highest contribution to the models for the IPM, MEM, TZP, and LVFX resistance of *P. aeruginosa*.

SUPPLEMENTARY FIGURE S1 The information about the filtering rules.

SUPPLEMENTARY FIGURE S2 Venn diagram.

SUPPLEMENTARY FIGURE S3 The relative abundance of AMR-associated genes for the IPM resistance between resistant samples and sensitive samples.

SUPPLEMENTARY FIGURE S4 The relative abundance of AMR-associated genes for the MEM resistance between resistant samples and sensitive samples.

SUPPLEMENTARY FIGURE S5 The relative abundance of AMR-associated genes for the TZP resistance between resistant samples and sensitive samples.

SUPPLEMENTARY FIGURE S6 The relative abundance of AMR-associated genes for the LVFX resistance of between resistant samples and sensitive samples.

## References

[ref1] Anderson S. E. Chin C. Y. Weiss D. S. Rather P. N. (2020). Copy number of an Integron-encoded antibiotic resistance locus regulates a virulence and opacity switch in *Acinetobacter baumannii* AB 5075. MBio 11:e02338–20. doi: 10.1128/mBio.02338-20 33024041 PMC7542366

[ref2] Bandyopadhyay D. Mukherjee M. (2022). Combination of bactericidal antibiotics and inhibitors of universal stress protein a (UspA): a potential therapeutic alternative against multidrug resistant *Escherichia coli* in urinary tract infections. J. Antibiot. 75, 21–28. doi: 10.1038/s41429-021-00477-4, PMID: 34526667

[ref3] Bernard P. Couturier M. (1992). Cell killing by the F plasmid CcdB protein involves poisoning of DNA-topoisomerase II complexes. J. Mol. Biol. 226, 735–745. doi: 10.1016/0022-2836(92)90629-X 1324324

[ref4] Cantalapiedra C. P. Hernández-Plaza A. Letunic I. Bork P. Huerta-Cepas J. (2021). Egg NOG-mapper v2: functional annotation, Orthology assignments, and domain prediction at the metagenomic scale. Mol. Biol. Evol. 38, 5825–5829. doi: 10.1093/molbev/msab293, PMID: 34597405 PMC8662613

[ref5] Castillo Y. P. Pérez M. A. (2008). Bacterial beta-ketoacyl-acyl carrier protein synthase III (FabH): an attractive target for the design of new broad-spectrum antimicrobial agents. Mini-Rev. Med. Chem. 8, 36–45. doi: 10.2174/138955708783331559, PMID: 18220983

[ref6] Chegini Z. Khoshbayan A. Taati Moghadam M. Farahani I. Jazireian P. Shariati A. (2020). Bacteriophage therapy against *Pseudomonas aeruginosa* biofilms: a review. Ann. Clin. Microbiol. Antimicrob. 19:45. doi: 10.1186/s12941-020-00389-5, PMID: 32998720 PMC7528332

[ref7] Chen S. Zhou Y. Chen Y. Gu J. (2018). Fastp: an ultra-fast all-in-one FASTQ preprocessor. Bioinformatics 34, i884–i890. doi: 10.1093/bioinformatics/bty560, PMID: 30423086 PMC6129281

[ref8] Chevalier S. Bouffartigues E. Bodilis J. Maillot O. Lesouhaitier O. Feuilloley M. G. J.. (2017). Structure, function and regulation of *Pseudomonas aeruginosa* porins. FEMS Microbiol. Rev. 41, 698–722. doi: 10.1093/femsre/fux020 28981745

[ref9] Chirakul S. Somprasong N. Norris M. H. Wuthiekanun V. Chantratita N. Tuanyok A.. (2019). *Burkholderia pseudomallei* acquired ceftazidime resistance due to gene duplication and amplification. Int. J. Antimicrob. Agents 53, 582–588. doi: 10.1016/j.ijantimicag.2019.01.003, PMID: 30639528

[ref10] Corral J. Pérez-Varela M. Sánchez-Osuna M. Cortés P. Barbé J. Aranda J. (2021). Importance of twitching and surface-associated motility in the virulence of *Acinetobacter baumannii*. Virulence 12, 2201–2213. doi: 10.1080/21505594.2021.1950268, PMID: 34515614 PMC8451467

[ref11] Cortes-Lara S. Barrio-Tofiño E. D. López-Causapé C. Oliver A. (2021). Predicting *Pseudomonas aeruginosa* susceptibility phenotypes from whole genome sequence resistome analysis. Clin. Microbiol. Infect. 27, 1631–1637. doi: 10.1016/j.cmi.2021.05.011 34015532

[ref12] da Cruz Nizer W. S. Adams M. E. Inkovskiy V. Beaulieu C. Overhage J. (2023). The secondary metabolite hydrogen cyanide protects *Pseudomonas aeruginosa* against sodium hypochlorite-induced oxidative stress. Front. Microbiol. 14:1294518. doi: 10.3389/fmicb.2023.1294518, PMID: 38033579 PMC10687435

[ref13] Doss V. A. Parvathi S. Raju B. A. Devi N. A. (2004). Evaluation on the use of beta-lactamase and aminoglycoside modifying enzyme gene sequences as markers for the early detection of antibiotic resistance profile of *Pseudomonas aeruginosa*. Dis. Markers 20, 317–323. doi: 10.1155/2004/690980, PMID: 15665392 PMC3839280

[ref14] Duvernay C. Coulange L. Dutilh B. Dubois V. Quentin C. Arpin C. (2011). Duplication of the chromosomal blaSHV-11 gene in a clinical hypermutable strain of *Klebsiella pneumoniae*. Microbiology 157, 496–503. doi: 10.1099/mic.0.043885-0 20966089

[ref15] Fajardo A. Martínez-Martín N. Mercadillo M. Galán J. C. Ghysels B. Matthijs S.. (2008). The neglected intrinsic resistome of bacterial pathogens. PLoS One 3:e1619. doi: 10.1371/journal.pone.0001619, PMID: 18286176 PMC2238818

[ref16] Fernández-Barat L. Ferrer M. De Rosa F. Gabarrús A. Esperatti M. Terraneo S.. (2017). Intensive care unit-acquired pneumonia due to *Pseudomonas aeruginosa* with and without multidrug resistance. J. Inf. Secur. 74, 142–152. doi: 10.1016/j.jinf.2016.11.008 27865895

[ref17] Greenblum S. Carr R. Borenstein E. (2015). Extensive strain-level copy-number variation across human gut microbiome species. Cell 160, 583–594. doi: 10.1016/j.cell.2014.12.038, PMID: 25640238 PMC4507803

[ref18] Hoang M. T. V. Irinyi L. Hu Y. Schwessinger B. Meyer W. (2021). Long-reads-based metagenomics in clinical diagnosis with a special focus on fungal infections. Front. Microbiol. 12:708550. doi: 10.3389/fmicb.2021.708550 35069461 PMC8770865

[ref19] Hu X. Zhao Y. (2023). Novel clinical mNGS-based machine learning model for rapid antimicrobial susceptibility testing of *Acinetobacter baumannii*. J. Clin. Microbiol. 61:e0180522. doi: 10.1128/jcm.01805-22 37022167 PMC10204632

[ref20] Hyun J. C. Kavvas E. S. (2020). Machine learning with random subspace ensembles identifies antimicrobial resistance determinants from pan-genomes of three pathogens. PLoS Comput. Biol. 16:e1007608. doi: 10.1371/journal.pcbi.1007608 32119670 PMC7067475

[ref21] Kaleta M. F. Petrova O. E. Zampaloni C. Garcia-Alcalde F. Parker M. Sauer K. (2022). A previously uncharacterized gene, PA 2146, contributes to biofilm formation and drug tolerance across the ɣ-Proteobacteria. NPJ Biofilms Microb. 8:54. doi: 10.1038/s41522-022-00314-y, PMID: 35798749 PMC9262955

[ref22] Kaleta M. F. Sauer K. (2023). Moa B1 homologs contribute to biofilm formation and motility by Pseudomonas aeruginosa and *Escherichia coli*. J. Bacteriol. 205:e0000423. doi: 10.1128/jb.00004-23, PMID: 37098964 PMC10210980

[ref23] Khaledi A. Weimann A. (2020). Predicting antimicrobial resistance in *Pseudomonas aeruginosa* with machine learning-enabled molecular diagnostics. EMBO Mol. Med. 12:e10264. doi: 10.15252/emmm.201910264, PMID: 32048461 PMC7059009

[ref24] Kim J. Greenberg D. E. (2020). VAMPr: VAriant mapping and prediction of antibiotic resistance via explainable features and machine learning. PLoS Comput. Biol. 16:e1007511. doi: 10.1371/journal.pcbi.1007511 31929521 PMC7015433

[ref25] Klockgether J. Cramer N. Wiehlmann L. Davenport C. F. Tümmler B. (2011). *Pseudomonas aeruginosa* genomic structure and diversity. Front. Microbiol. 2:150. doi: 10.3389/fmicb.2011.00150 21808635 PMC3139241

[ref26] Kvint K. Nachin L. Diez A. Nyström T. (2003). The bacterial universal stress protein: function and regulation. Curr. Opin. Microbiol. 6, 140–145. doi: 10.1016/S1369-5274(03)00025-0 12732303

[ref27] Langmead B. Salzberg S. L. (2012). Fast gapped-read alignment with bowtie 2. Nat. Methods 9, 357–359. doi: 10.1038/nmeth.1923, PMID: 22388286 PMC3322381

[ref28] Lewis K. (2010). Persister cells. Ann. Rev. Microbiol. 64, 357–372. doi: 10.1146/annurev.micro.112408.134306 20528688

[ref29] Lister P. D. Wolter D. J. Hanson N. D. (2009). Antibacterial-resistant *Pseudomonas aeruginosa*: clinical impact and complex regulation of chromosomally encoded resistance mechanisms. Clin. Microbiol. Rev. 22, 582–610. doi: 10.1128/CMR.00040-09, PMID: 19822890 PMC2772362

[ref30] Liu B. Gao J. Liu X. F. Rao G. (2023). Direct prediction of carbapenem resistance in *Pseudomonas aeruginosa* by whole genome sequencing and metagenomic sequencing. J. Clin. Microbiol. 61:e0061723. doi: 10.1128/jcm.00617-23 37823665 PMC10662344

[ref31] Maddamsetti R. Yao Y. Wang T. Gao J. Huang V. T. Hamrick G. S.. (2024). Duplicated antibiotic resistance genes reveal ongoing selection and horizontal gene transfer in bacteria. Nat. Commun. 15:1449. doi: 10.1038/s41467-024-45638-9, PMID: 38365845 PMC10873360

[ref32] Maladan Y. Krismawati H. Wahyuni T. Tanjung R. Awaludin K. Audah K. A.. (2021). The whole-genome sequencing in predicting *Mycobacterium tuberculosis* drug susceptibility and resistance in Papua, Indonesia. BMC Genomics 22:844. doi: 10.1186/s12864-021-08139-3, PMID: 34802420 PMC8607662

[ref33] McGann P. Courvalin P. Snesrud E. Clifford R. J. Yoon E. J. Onmus-Leone F.. (2014). Amplification of aminoglycoside resistance gene aphA1 in *Acinetobacter baumannii* results in tobramycin therapy failure. MBio 5:e00915. doi: 10.1128/mBio.00915-14, PMID: 24757213 PMC3994513

[ref34] Murray C. J. L. Ikuta K. S. Sharara F. Swetschinski L. Robles Aguilar G. Gray A.. (2022). Global burden of bacterial antimicrobial resistance in 2019: a systematic analysis. Lancet 399, 629–655. doi: 10.1016/S0140-6736(21)02724-0, PMID: 35065702 PMC8841637

[ref35] Oguri T. Kwon Y. Woo J. K. K. Prehna G. Lee H. Ning M.. (2019). A family of small intrinsically disordered proteins involved in flagellum-dependent motility in *Salmonella enterica*. J. Bacteriol. 201:e00415–18. doi: 10.1128/JB.00415-18 30373755 PMC6304668

[ref36] Parsons J. B. Yao J. Frank M. W. Rock C. O. (2015). FabH mutations confer resistance to FabF-directed antibiotics in *Staphylococcus aureus*. Antimicrob. Agents Chemother. 59, 849–858. doi: 10.1128/AAC.04179-14, PMID: 25403676 PMC4335864

[ref37] Poole K. (2002). Outer membranes and efflux: the path to multidrug resistance in gram-negative bacteria. Curr. Pharm. Biotechnol. 3, 77–98. doi: 10.2174/1389201023378454, PMID: 12022261

[ref38] Qin S. Xiao W. Zhou C. Pu Q. Deng X. Lan L. (2022). *Pseudomonas aeruginosa*: pathogenesis, virulence factors, antibiotic resistance, interaction with host, technology advances and emerging therapeutics. Signal Transduct. Target. Therapy 7:199. doi: 10.1038/s41392-022-01056-1 PMC9233671 35752612

[ref39] Raina J. B. Fernandez V. Lambert B. Stocker R. Seymour J. R. (2019). The role of microbial motility and chemotaxis in symbiosis. Nat. Rev. Microbiol. 17, 284–294. doi: 10.1038/s41579-019-0182-9 30923350

[ref40] Reynolds D. Kollef M. (2021). The epidemiology and pathogenesis and treatment of *Pseudomonas aeruginosa* infections: an update. Drugs 81, 2117–2131. doi: 10.1007/s40265-021-01635-6 34743315 PMC8572145

[ref41] Ruppé E. d'Humières C. Armand-Lefèvre L. (2022). Inferring antibiotic susceptibility from metagenomic data: dream or reality? Clin. Microbiol. Infect. 28, 1225–1229. doi: 10.1016/j.cmi.2022.04.017 35551982

[ref42] Shanmugakani R. K. Srinivasan B. Glesby M. J. Westblade L. F. Cárdenas W. B. Raj T.. (2020). Current state of the art in rapid diagnostics for antimicrobial resistance. Lab Chip 20, 2607–2625. doi: 10.1039/D0LC00034E, PMID: 32644060 PMC7428068

[ref43] Shen W. Le S. Li Y. Hu F. (2016). Seq kit: a cross-platform and ultrafast toolkit for FASTA/Q file manipulation. PLoS One 11:e0163962. doi: 10.1371/journal.pone.0163962, PMID: 27706213 PMC5051824

[ref44] Tenover F. C. Nicolau D. P. Gill C. M. (2022). Carbapenemase-producing *Pseudomonas aeruginosa*-an emerging challenge. Emerg. Microb. Infect. 11, 811–814. doi: 10.1080/22221751.2022.2048972, PMID: 35240944 PMC8920394

[ref45] Thacharodi A. Lamont I. L. (2022). Aminoglycoside-modifying enzymes are sufficient to make Pseudomonas aeruginosa clinically resistant to key antibiotics. Antibiotics 11:884. doi: 10.3390/antibiotics11070884 35884138 PMC9312099

[ref46] Unterholzner S. J. Hailer B. Poppenberger B. Rozhon W. (2013). Characterisation of the stbD/E toxin-antitoxin system of pEP36, a plasmid of the plant pathogen *Erwinia pyrifoliae*. Plasmid 70, 216–225. doi: 10.1016/j.plasmid.2013.04.002, PMID: 23632277

[ref47] Wadhwa N. Berg H. C. (2022). Bacterial motility: machinery and mechanisms. Nat. Rev. Microbiol. 20, 161–173. doi: 10.1038/s41579-021-00626-4 34548639

[ref48] Xu Y. Jing Y. Hu L. Cheng Q. Gao H. Zhang Z.. (2022). IncFIB-4.1 and IncFIB-4.2 single-replicon plasmids: small backbones with large accessory regions. Infect. Drug Resist. 15, 1191–1203. doi: 10.2147/IDR.S332949, PMID: 35345473 PMC8957301

[ref49] Yan G. Liu J. Chen W. Chen Y. Cheng Y. Tao J.. (2021). Metagenomic next-generation sequencing of bloodstream microbial cell-free nucleic acid in children with suspected Sepsis in pediatric intensive care unit. Front. Cell. Infect. Microbiol. 11:665226. doi: 10.3389/fcimb.2021.665226, PMID: 34504805 PMC8421769

[ref50] Yao Y. Maddamsetti R. Weiss A. Ha Y. Wang T. Wang S.. (2022). Intra-and interpopulation transposition of mobile genetic elements driven by antibiotic selection. Nat. Ecol. Evol. 6, 555–564. doi: 10.1038/s41559-022-01705-2, PMID: 35347261 PMC12520065

[ref51] Yokoyama K. Doi Y. Yamane K. Kurokawa H. Shibata N. Shibayama K.. (2003). Acquisition of 16S rRNA methylase gene in *Pseudomonas aeruginosa*. Lancet 362, 1888–1893. doi: 10.1016/S0140-6736(03)14959-8, PMID: 14667745

[ref52] Yuan Y. Sachdeva M. Leeds J. A. Meredith T. C. (2012). Fatty acid biosynthesis in *Pseudomonas aeruginosa* is initiated by the FabY class of β-ketoacyl acyl carrier protein synthases. J. Bacteriol. 194, 5171–5184. doi: 10.1128/JB.00792-12, PMID: 22753059 PMC3457228

[ref53] Zeevi D. Korem T. Godneva A. Bar N. Kurilshikov A. Lotan-Pompan M.. (2019). Structural variation in the gut microbiome associates with host health. Nature 568, 43–48. doi: 10.1038/s41586-019-1065-y 30918406

