# Supplementary figures and images for: Direct prediction of antimicrobial resistance in Pseudomonas aeruginosa by metagenomic next-generation sequencing
Source: Front Microbiol. 2024 Jun 6;15:1413434. doi: 10.3389/fmicb.2024.1413434 (PMC11187003; doi:10.3389/fmicb.2024.1413434)

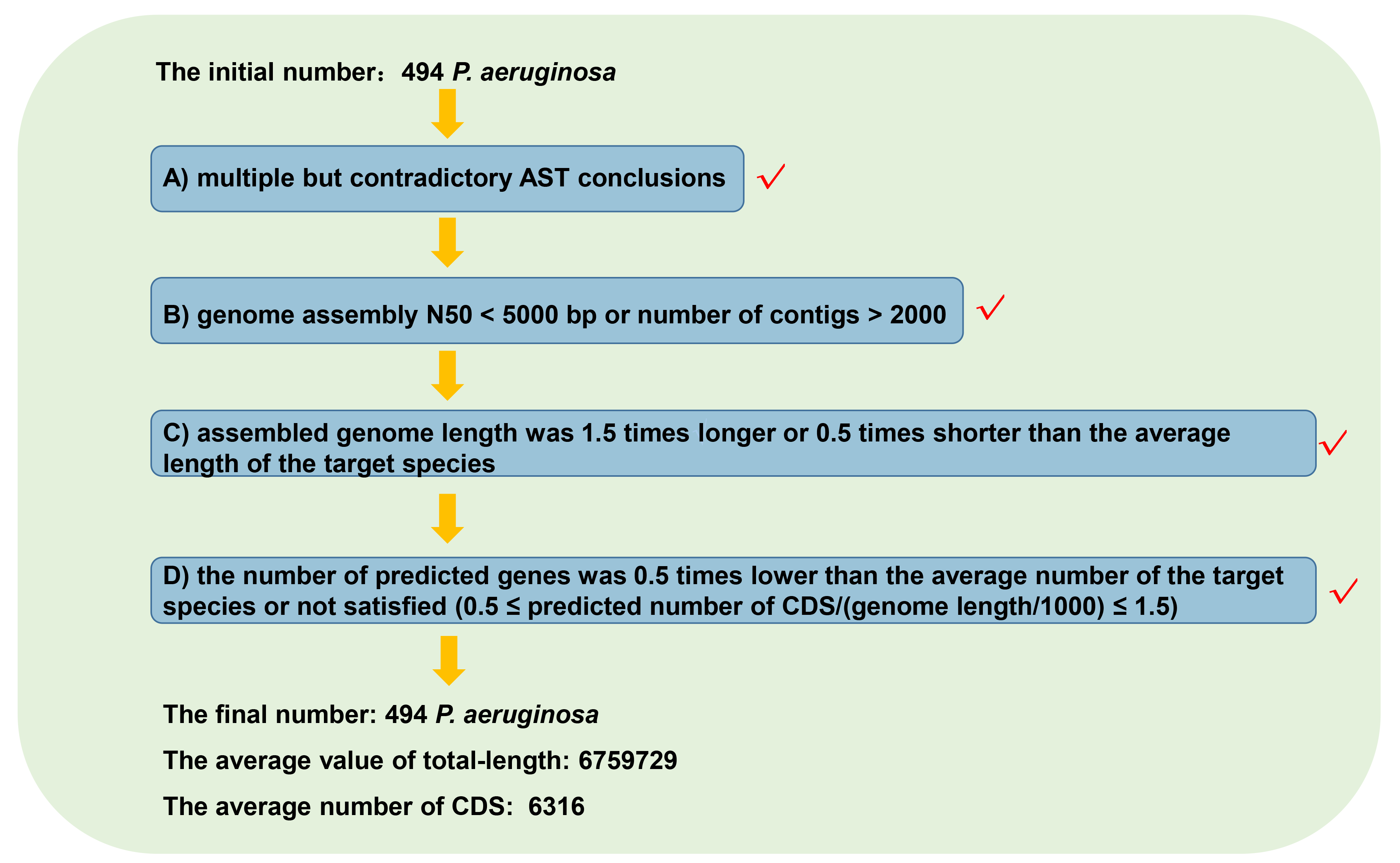

Supplement: SUPPLEMENTARY FIGURE S1 — The information about the filtering rules. [file Image_1.TIF]

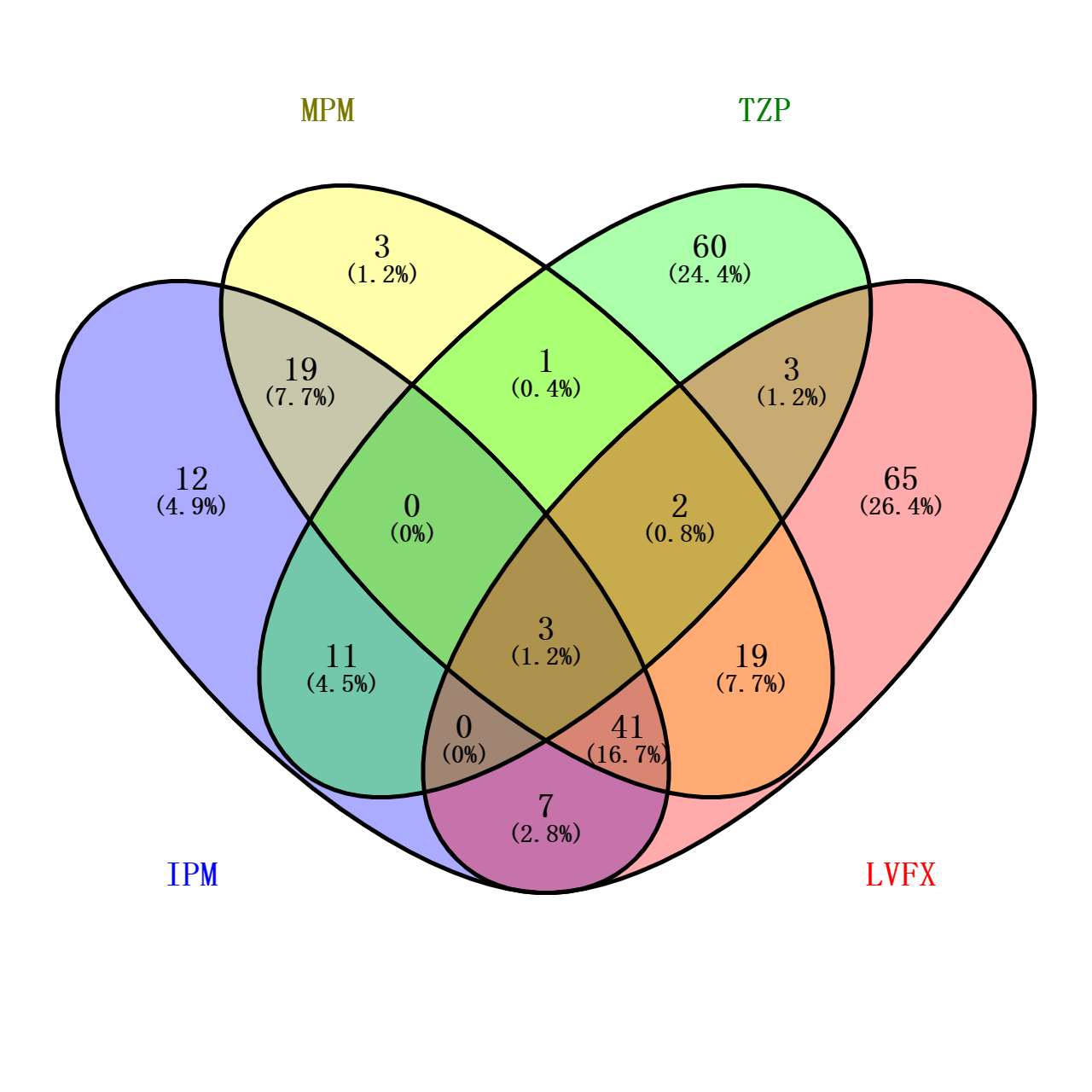

Supplement: SUPPLEMENTARY FIGURE S2 — Venn diagram. [file Image_2.TIF]

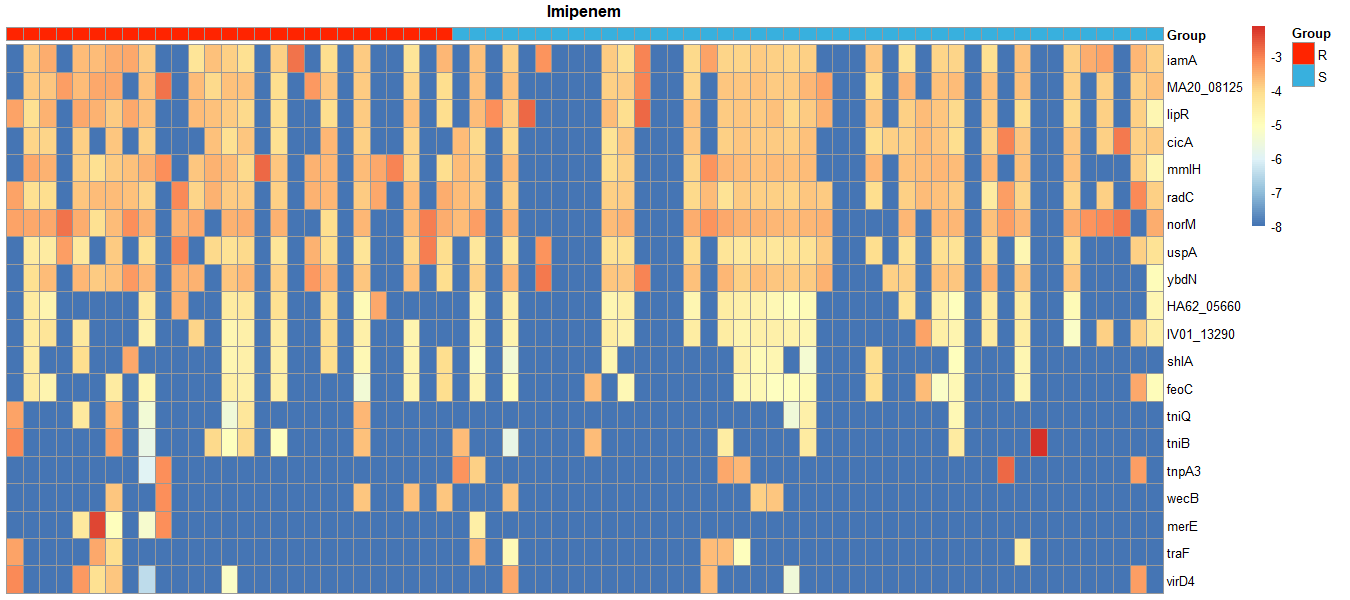

Supplement: SUPPLEMENTARY FIGURE S3 — The relative abundance of AMR-associated genes for the IPM resistance between resistant samples and sensitive samples. [file Image_3.TIFF]

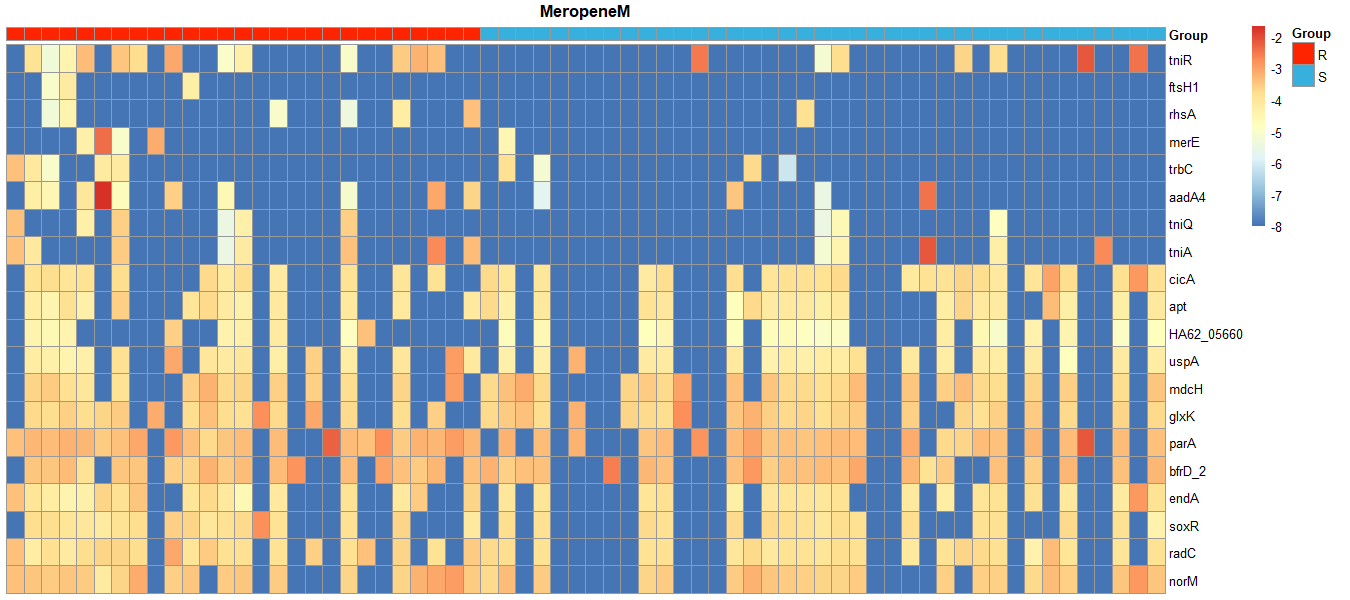

Supplement: SUPPLEMENTARY FIGURE S4 — The relative abundance of AMR-associated genes for the MEM resistance between resistant samples and sensitive samples. [file Image_4.TIFF]

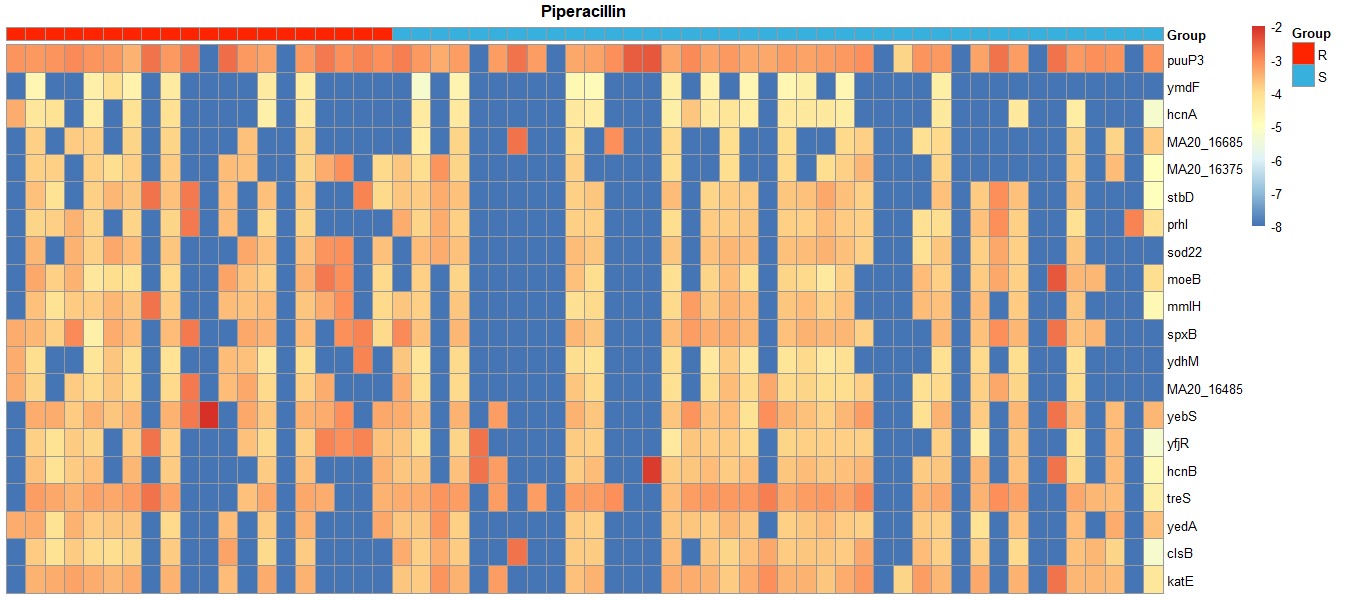

Supplement: SUPPLEMENTARY FIGURE S5 — The relative abundance of AMR-associated genes for the TZP resistance between resistant samples and sensitive samples. [file Image_5.TIFF]

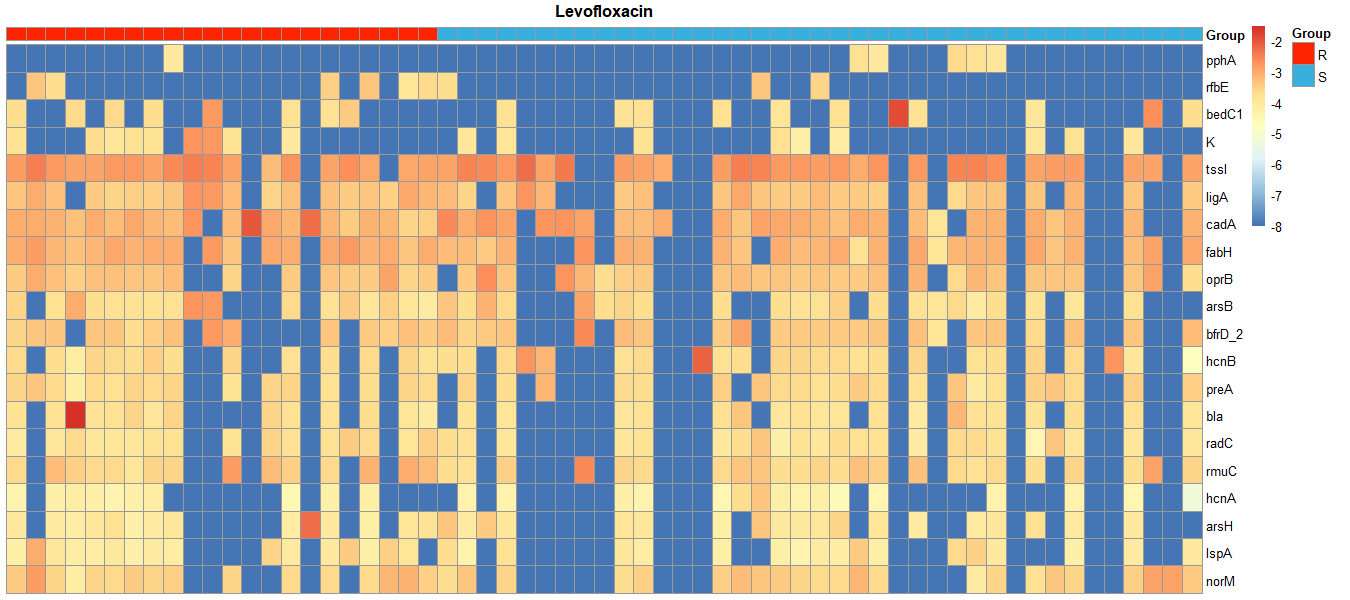

Supplement: SUPPLEMENTARY FIGURE S6 — The relative abundance of AMR-associated genes for the LVFX resistance of between resistant samples and sensitive samples. [file Image_6.TIFF]
